# COVID-19 Case Investigation and Contact Tracing in the US, 2020

**DOI:** 10.1001/jamanetworkopen.2021.15850

**Published:** 2021-06-03

**Authors:** R. Ryan Lash, Patrick K. Moonan, Brittany L. Byers, Robert A. Bonacci, Kimberly E. Bonner, Matthew Donahue, Catherine V. Donovan, Heather N. Grome, Julia M. Janssen, Reed Magleby, Heather P. McLaughlin, James S. Miller, Caroline Q. Pratt, Jonathan Steinberg, Kate Varela, Greta L. Anschuetz, Paul R. Cieslak, Veronica Fialkowski, Aaron T. Fleischauer, Clay Goddard, Sara Jo Johnson, Michelle Morris, Jill Moses, Allison Newman, Lauren Prinzing, Alana C. Sulka, Puthiery Va, Matthew Willis, John E. Oeltmann

**Affiliations:** 1 Epidemic Intelligence Service, Centers for Disease Control and Prevention, Atlanta, Georgia; 2 COVID-19 Response Team, Centers for Disease Control and Prevention, Atlanta, Georgia; 3 Public Health Division, Oregon Health Authority, Portland; 4 Nebraska Department of Health and Human Services, Lincoln; 5 North Carolina Department of Health and Human Services, Raleigh; 6 Tennessee Department of Health, Nashville; 7 New Jersey Department of Health, Trenton; 8 Washington State Department of Health, Tumwater; 9 South Dakota State Health Department, Sioux Falls; 10 Vermont Department of Health, Burlington; 11 Career Epidemiology Field Officer Program, Centers for Disease Control and Prevention, Atlanta, Georgia; 12 Springfield-Greene County Health Department, Springfield, Missouri; 13 Polk County Health Center, Des Moines, Iowa; 14 Chinle Indian Health Service Unit, Chinle, Arizona; 15 Gwinnett, Newton, Rockdale Counties Health Departments, Lawrenceville, Georgia; 16 Marin County Public Health, San Rafael, California

## Abstract

**Importance:**

Contact tracing is a multistep process to limit SARS-CoV-2 transmission. Gaps in the process result in missed opportunities to prevent COVID-19.

**Objective:**

To quantify proportions of cases and their contacts reached by public health authorities and the amount of time needed to reach them and to compare the risk of a positive COVID-19 test result between contacts and the general public during 4-week assessment periods.

**Design, Setting, and Participants:**

This cross-sectional study took place at 13 health departments and 1 Indian Health Service Unit in 11 states and 1 tribal nation. Participants included all individuals with laboratory-confirmed COVID-19 and their named contacts. Local COVID-19 surveillance data were used to determine the numbers of persons reported to have laboratory-confirmed COVID-19 who were interviewed and named contacts between June and October 2020.

**Main Outcomes and Measures:**

For contacts, the numbers who were identified, notified of their exposure, and agreed to monitoring were calculated. The median time from index case specimen collection to contact notification was calculated, as were numbers of named contacts subsequently notified of their exposure and monitored. The prevalence of a positive SARS-CoV-2 test among named and tested contacts was compared with that jurisdiction’s general population during the same 4 weeks.

**Results:**

The total number of cases reported was 74 185. Of these, 43 931 (59%) were interviewed, and 24 705 (33%) named any contacts. Among the 74 839 named contacts, 53 314 (71%) were notified of their exposure, and 34 345 (46%) agreed to monitoring. A mean of 0.7 contacts were reached by telephone by public health authorities, and only 0.5 contacts per case were monitored. In general, health departments reporting large case counts during the assessment (≥5000) conducted smaller proportions of case interviews and contact notifications. In 9 locations, the median time from specimen collection to contact notification was 6 days or less. In 6 of 8 locations with population comparison data, positive test prevalence was higher among named contacts than the general population.

**Conclusions and Relevance:**

In this cross-sectional study of US local COVID-19 surveillance data, testing named contacts was a high-yield activity for case finding. However, this assessment suggests that contact tracing had suboptimal impact on SARS-CoV-2 transmission, largely because 2 of 3 cases were either not reached for interview or named no contacts when interviewed. These findings are relevant to decisions regarding the allocation of public health resources among the various prevention strategies and for the prioritization of case investigations and contact tracing efforts.

## Key Points

**Question:**

What proportion of persons with laboratory-confirmed COVID-19 and their contacts are reached for case investigation and contact tracing?

**Findings:**

In this surveillance-based, cross-sectional study, 2 of 3 individuals with COVID-19 were either not reached for interview or named no contacts when interviewed. A mean of 0.7 contacts were reached by telephone by public health authorities, and only 0.5 contacts per case were monitored, a lower rate than needed to overcome the estimated global SARS-CoV-2 reproductive number.

**Meaning:**

The findings of this study suggest that current contact tracing practice had suboptimal impact on SARS-CoV-2 transmission.

## Teaser

This cross-sectional study examines proportions of individuals with confirmed COVID-19 cases and their contacts reached by public health authorities and the amount of time needed to reach them and compares the risk of a positive test result between contacts and the general public.

## Introduction

During 2020, mitigation of SARS-CoV-2, the virus that causes COVID-19, largely depended on nonpharmaceutical interventions, such as physical distancing, hand hygiene, universal masking, isolation, and quarantine.^1,2,3,4,5^ Contact tracing and quarantine of exposed contacts are intended to limit the number of infected contacts who propagate transmission.^6,7,8^ Contact tracing is a resource-intensive, multistep process^9,10^ with many potential steps at which persons with COVID-19 or their contacts might be missed or notified too late of their infection or exposure. Presymptomatic and asymptomatic transmission is a major challenge to contact tracing,^11,12,13^ as are passive symptom monitoring policies that place the responsibility to seek medical attention on the person experiencing symptoms.^14^ Moreover, potential stigma and lack of trust in government officials might undermine contact tracing efforts.^15,16^ Evaluations of contact tracing for other airborne diseases suggest that name-based contact tracing might fail to identify important contacts, even within households.^17,18^

Health departments (HDs) across the US use contact tracing to reduce transmission of infectious diseases.^19,20^ COVID-19 presents new challenges, most notably the high prevalence of disease in many jurisdictions and the emergence of more transmissible virus variants.^21^ The National Academies of Sciences, Engineering, and Medicine categorized 3 main behavioral challenges during the COVID-19 contact tracing process: lack of response to telephone calls from local public health officials, reluctance to disclose information because of lack of trust in government, and unwillingness to share names of potentially exposed persons resulting from fear of stigma or disinclination to subject others to quarantine restrictions.^22^ It should also be recognized that HDs may not have been adequately resourced to reach all cases and contacts. Recent reports^23,24^ corroborate the contact tracing challenges HDs encountered during periods of high COVID-19 incidence. Despite the aggressive efforts by HD staff in North Carolina, many cases were not reached for interview, and many who were reached named no contacts.^23^ To assess the completeness and timeliness of COVID-19 contact tracing during 2020, we worked with HDs to measure the number and proportion of persons reached at several critical steps along the case investigation and contact tracing cascade (eFigure in Supplement 1).

## Methods

This cross-sectional study examined routinely collected COVID-19 case investigation and contact tracing data from existing local databases. This activity was determined to be public health surveillance as defined in 45 CFR 46.102(l). (US Department of Health and Human Services, Title 45 Code of Federal Regulations 46, Protection of Human Subjects); thus, it was not submitted for institutional review board approval and informed consent was not needed. This study follows the Strengthening the Reporting of Observational Studies in Epidemiology (STROBE) reporting guideline for cross-sectional studies.^25^

Thirteen HDs and 1 Indian Health Service Unit in 11 states and 1 tribal nation collaborated in asynchronous 4-week assessments. Assessment periods occurred from June 1 to October 31, 2020. This convenience sample of participating HDs included counties, health districts (several adjacent counties), and entire states, as well as 1 Indian Health Service Unit, together representing different regions of the US and different population densities. HDs were selected to maximize variation in geography, population size, and population density, along with the availability and willingness to share data. HDs were categorized according to US Census Bureau Regions. Daily case count data^26^ were used to calculate mean weekly incidence per 100 000 persons^27^ during each HD’s 4-week assessment period.

Using routinely collected COVID-19 case investigation and contact tracing data from existing local databases, we quantified how many persons with laboratory-confirmed COVID-19 were reported for public health surveillance, how many were interviewed, and how many named contacts. For contacts, we calculated the total number who were identified, notified of their exposure, and monitored for COVID-19 symptoms.

Because not every person with laboratory-confirmed COVID-19 could be reached for an interview, and not all interviewed persons named contacts, we compared each HD’s total number of elicited contacts with an estimate of the expected number had all persons with COVID-19 been interviewed and named contacts. For each HD, the expected number of contacts per case was based on the local mean number of named contacts among the persons the HD interviewed who did name contacts. This local mean was then multiplied by the total number of reported cases during that HD’s 4-week assessment period.

### Statistical Analysis

Epidemiological trends for each study locations were characterized on the basis of the mean weekly percentage change in incidence over an 8-week period (encompassing the 4-week assessment period and the 4 weeks prior). We used regression analysis with Joinpoint statistical software version 4.8.0.1 (National Cancer Institute)^28^ and 95% CIs to test the rate of change for each trend. The weekly percentage change was significantly different from 0 at α = .05.

Four HDs provided race and ethnicity data for persons with COVID-19. We used prevalence ratios (PRs) and 95% CIs to examine whether contact elicitation differed across racial and ethnic groups. We also calculated the median, interquartile range (IQR), and range for the time from index case specimen collection to when test results were reported to the HD and to when the HD notified contacts of their exposure. Eight HDs provided SARS-CoV-2 test positivity data for named contacts. We compared the prevalence of a positive SARS-CoV-2 test result among named contacts with that in the jurisdiction’s general population using a PR and 95% CI. The prevalence of a positive test in the general population was calculated on the basis of the total number of positive polymerase chain reaction test results among the total reported test results in each location during the assessment period.

## Results

### COVID-19 Incidence in the 14 HDs

**Figure 1.**
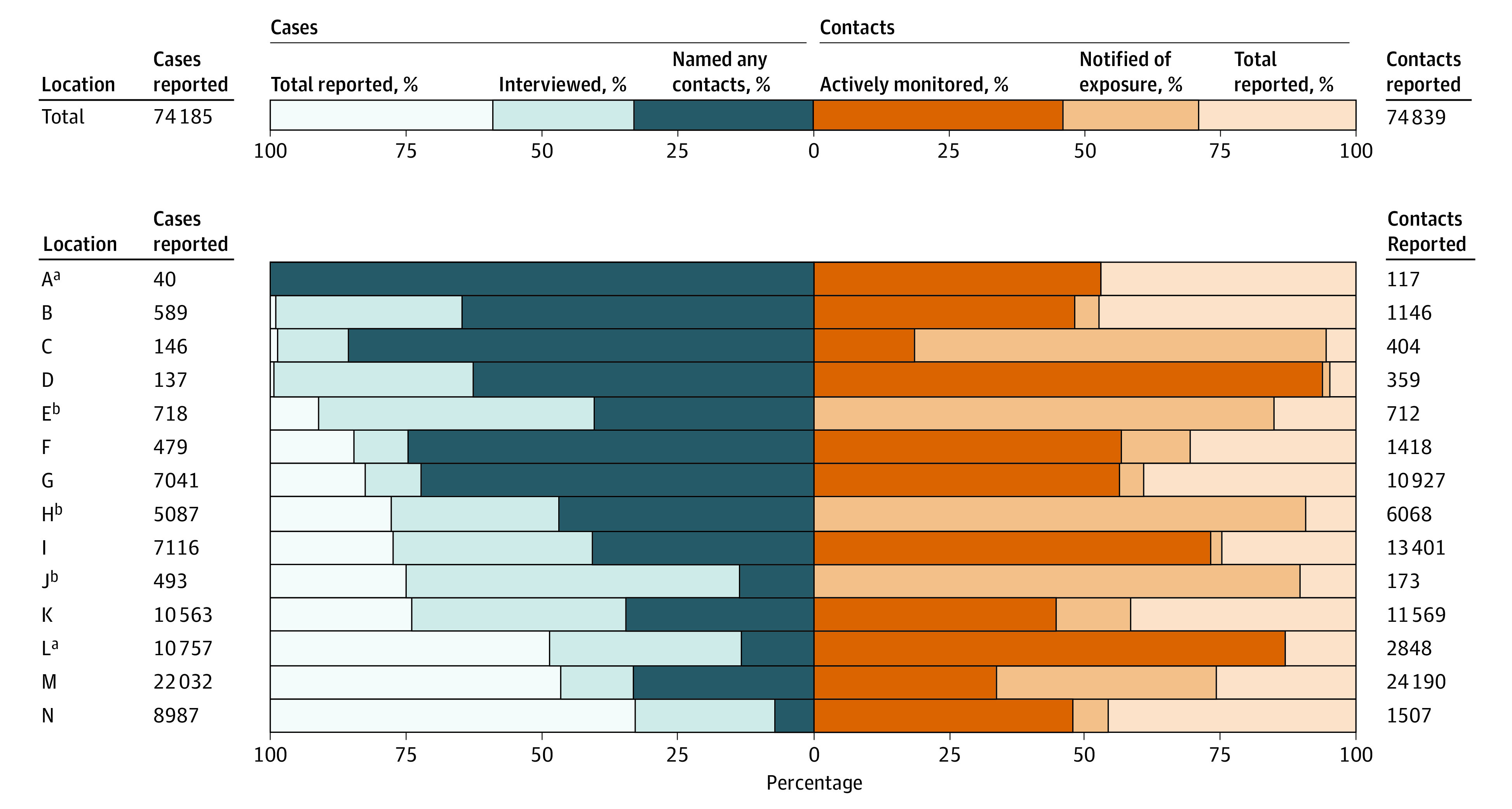
Key Case Investigation and Contact Tracing Assessment Metrics, June to October 2020 The cumulative totals from all 14 locations were 74 185 individuals with reported and confirmed COVID-19 (59% were interviewed, and 33% named any contacts), resulting in 74 839 reported contacts (71% were notified of exposure, and 46% were actively monitored) (top panel). Locations have been sorted in descending order according to the proportion of persons reported as having laboratory-confirmed SARS-CoV-2 infection who were interviewed (bottom panel). ^a^All of the contacts who were reached and notified of their exposure also agreed to be actively monitored. ^b^Active monitoring was not performed in these locations.

Seven HDs reported more than 5000 COVID-19 cases during the 4-week assessment; the remaining 7 HDs reported fewer than 1000 cases (Figure 1). The mean weekly incidence varied widely (range, 6.3-621.9 cases per 100 000 persons) during the assessment period. Trends in COVID-19 incidence significantly increased in 7 locations (mean weekly change in incidence, 6.3% [95% CI, 0.7% to 12.2%] for location B, 67.0% [95% CI, 5.3% to 165%] for location D, 28.3% [95% CI, 4.5% to 57.6%] for location F, 26.0% [95% CI, 20% to 32.2%] for location I, 18.6% [95% CI, 3.3% to 36%] for location L, 24.8% [95% CI, 21.6% to 28.1%] for location M, and 21.4% [95% CI, 3.4% to 42.5%] for location N) and significantly decreased in 3 locations (mean weekly change in incidence, −20.9% [95% CI, −27.1% to −14.1%] for location A, −17.8% [95% CI, −24.2% to −10.9%] for location E, and −3.6% [95% CI, −7.0% to −0.1%] for location G) (Table 1).

**Table 1.** Characteristics of the 14 Participating Health Departments

Location	US Census Region	Health department type	Estimated population, persons, No.^a^	Population per square mile, persons, No.	Dates in 2020 of 4-wk analysis period	Mean weekly incidence^b^	Weekly change in incidence, mean (95% CI), %^c^
A	West	Local	30 781	11	August 23-September 19	30.9	−20.9 (−27.1 to −14.1)^d^
B	South	Local	143 667	184	June 15-July 12	97.1	6.3 (0.7 to 12.2)^d^
C	Northeast	State	623 989	68	July 26-August 22	6.3	0.0 (−10.1 to 11.2)
D	Midwest	Local	32 149	51	June 28-July 25	106.5	67.0 (5.3 to 165)^d^
E	West	Local	258 826	497	July 30-August 26	121.2	−17.8 (−24.2 to −10.9)^d^
F	Midwest	Local	293 086	434	June 21-July 18	32.2	28.3 (4.5 to 57.6)^d^
G	West	State	4 217 737	44	August 1-August 31	44.3	−3.6 (−7.0 to −0.1)^d^
H	Midwest	State	1 934 408	24	August 1-August 31	95.1	6.7 (−5.4 to 20.4)
I	South	Local	1 110 356	2120	June 1-June 30	144.5	26.0 (20 to 32.2)^d^
J	West	Local	120 629	25	June 15-July 12	99.3	19.7 (−14.3 to 67.2)
K	Northeast	State	8 882 190	1196	August 2-August 29	29.3	1.3 (−7.1 to 10.4)
L	South	Local	694 144	1377	July 1-July 31	317.6	18.6 (3.3 to 36)^d^
M	Midwest	State	884 659	11	October 4-October 31	621.9	24.8 (21.6 to 28.1)^d^
N	South	Local	1 138 890	1368	July 5-August 1	208.6	21.4 (3.4 to 42.5)^d^

^a^ US Census 2019 population estimates are available at https://www.census.gov/data.html.

^b^ Refers to mean weekly cases per 100 000 persons during the 4-week assessment period.

^c^ The mean weekly percentage change in incidence was calculated over an 8-week period (ie, encompassing the 4-week assessment period and the 4 weeks prior).

^d^ Indicates that weekly percentage change is significantly different from 0 at the α = .05 level.

### Proportions of Persons With COVID-19 Who Received Case Interviews and Named Contacts

Among the total 74 185 persons across all locations reported to HDs as having laboratory-confirmed COVID-19, 43 931 (59%) received case interviews, and 24 705 (33%) named contacts (Figure 1 and eTable 1 in Supplement 1). However, of these 43 931 persons interviewed, nearly one-half, or 19 226 (44%), named no contacts.

Wide variations in key case investigation and contact tracing metrics were observed across the 14 locations. In rural location A, the mean weekly COVID-19 incidence was 30.9 cases per 100 000 persons and decreased by a mean of 20.9% per week during the study period (Table 1). This HD interviewed all 40 (100%) persons with COVID-19 and elicited contacts from all 40 (100%). In contrast, suburban location N had a mean weekly COVID-19 incidence of 208.6 cases per 100 000 persons, which increased by a mean of 21.4% per week (Table 1). This HD interviewed 2962 of 8987 persons with COVID-19 (33%) and elicited contacts from 631 (7%) of these (Figure 1 and eTable 1 in Supplement 1). Four HDs (B, J, M, and N) provided data on race and ethnicity of reported cases; the success of contact elicitation was similar across all racial and ethnic groups in those 4 HDs (eTable 2 in Supplement 1).

HDs in locations with higher case counts conducted proportionally fewer case interviews than HDs with lower case counts (Figure 1). Six of 7 HDs with more than 5000 cases were unable to conduct case interviews or elicit contacts for a majority of cases. In contrast, only 2 of 7 HDs with fewer than 1000 cases were unable to conduct case interviews or elicit contacts for the majority of cases. Locations with more than 5000 cases were locations with larger populations.

Public health informatics capacity varied across HDs. Three HDs relied on paper-based data systems for collecting and managing information, and 11 HDs used digital data systems.

### Proportions of Named Contacts Who Were Notified of Their Exposure and Monitored

HDs notified 53 314 of 74 839 named contacts (71%) of their COVID-19 exposure. The proportion of contacts notified of their exposure ranged from 53% to 95% (Figure 1 and eTable 1 in Supplement 1). A mean of 0.7 contacts were reached by telephone by public health authorities, and only 0.5 contacts per case were monitored.

Three HDs (locations E, H, and J) did not include active contact monitoring (eg, telephone, text message, or email inquiries from public health authorities regarding COVID-19 symptoms or other changes in health status). Eleven HDs offered active monitoring; among the 47 056 contacts notified of their exposure from these locations, 34 345 (73%) agreed to participate. Thus, 46% of all named contacts from all participating locations were actively monitored by public health authorities during their quarantine periods.

### Estimated Number of Missed Contacts

We estimated that an expected total of 218 389 named contacts would have been generated from the 74 185 persons with COVID-19, rather than the observed 74 839. Thus, we estimated that 143 550 contacts (66%) at risk of recent exposure to SARS-CoV-2 might have been missed.

### Timeliness of SARS-CoV-2 Diagnostic Test Results and Contact Notification

**Figure 2.**
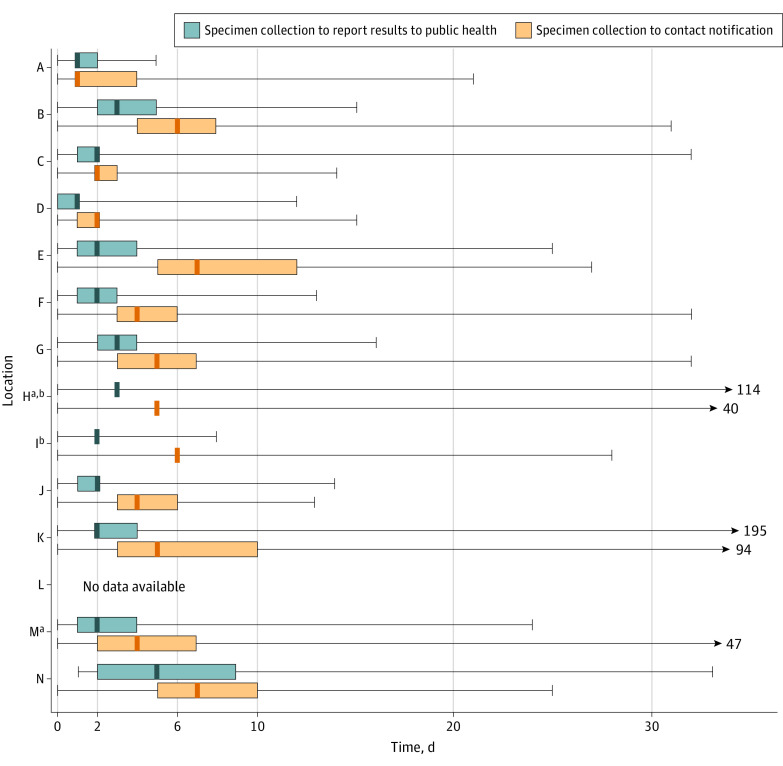
Timeliness of SARS-CoV-2 Diagnostic Test Results and Contact Notification, June to October 2020 Lines within bars denote medians, upper and lower bounds of the bars denote the interquartile ranges, and the error bars denote the full ranges. Timeliness data were not available from location L. ^a^Reported household contacts were notified during the index case investigation. ^b^The interquartile range was not available.

In the locations where timeliness could be assessed (all except location L) (Figure 2), the median time from index case specimen collection to positive test report to HD was 2 days (range, 1-5 days). In 9 locations, the median time from index case specimen collection to contact notification was 6 days or less.

### SARS-CoV-2 Test Positivity Among Named Contacts Compared With Positivity in Location’s General Population

The prevalence of a positive test result among contacts was available from 8 locations (Table 2). Compared with positive test prevalence in the general population during the assessment period, positive test prevalence among contacts was higher in 6 locations (PR, 5.8 [95% CI, 2.6-13.0] in location A; PR, 2.1 [95% CI, 1.7-2.6] in location B; PR, 5.1 [95% CI, 3.9-6.6] in location F; PR, 2.7 [95% CI, 2.4-2.9] in location L; and PR, 1.2 [95% CI, 1.2-1.3] in location M), and most notably in location C (PR, 17.6 [95% CI, 10.6-29.2]). In locations D (PR, 1.2 [95% CI, 0.7-1.8]) and N (PR, 0.9 [95% CI, 0.7-1.2]), the positive test prevalence among contacts was similar to that observed in the general population.

**Table 2.** SARS-CoV-2 Test Positivity Among Named Contacts Compared With Positivity in the General Population

Location	Identified contacts, No.	Contacts tested, No. (%)	Identified contact test positivity, No. (%)	General population test positivity, %	Prevalence ratio (95% CI)
A	117	64 (54.7)	8 (12.5)	2.4	5.8 (2.6-13.0)
B	1146	293 (25.6)	69 (23.5)	11.2	2.1 (1.7-2.6)
C	404	192 (47.5)	15 (7.8)	0.4	17.6 (10.6-29.2)
D	359	152 (42.3)	21 (13.8)	11.7	1.2 (0.7-1.8)
E	712	NA	NA	7.2	NA
F	1418	241 (16.9)	47 (19.5)	3.9	5.1 (3.9-6.6)
G	10 927	NA	842	NA	NA
H	6068	NA	NA	7.8	NA
I	13 401	NA	137	13.1	NA
J	173	NA	NA	NA	NA
K	11 569	NA	367	2.5	NA
L	2848	973 (34.2)	281 (28.9)	10.9	2.7 (2.4-2.9)
M	24 190	3418 (14.1)	680 (19.9)	16.7	1.2 (1.2-1.3)
N	1507	297 (19.7)	36 (12.1)	13.2	0.9 (0.7-1.2)

Abbreviation: NA, not applicable.

## Discussion

In this 14-location assessment, no contacts were reported for two-thirds of persons with laboratory-confirmed COVID-19 because they were either not reached for an interview or were interviewed and named no contacts. This assessment suggests that contact tracing activities were not sufficient for reducing SARS-CoV-2 transmission in most communities during June to October 2020. A mean of 0.7 contacts were reached by telephone by public health authorities, and only 0.5 contacts per case were monitored, a lower rate than needed to overcome the estimated global SARS-CoV-2 reproductive number, R_0_ (range, 2.2-8.9).^29^

On the other hand, when the contacts identified through case interviews were notified and tested, in most locations their SARS-CoV-2 test positivity was higher than that in the general population. Because contacts are known to have been recently exposed, we expect to find more undiagnosed COVID-19 cases among them, demonstrating that contact tracing can be beneficial when case interviews reveal names of important contacts. In the 4 locations that submitted data by race and ethnicity, naming 0 contacts appeared similar by racial and ethnic groups.

Populations, cultures, public health programs, and COVID-19 transmission intensity all varied widely by location and likely were associated with HDs’ ability to reach cases and elicit contacts. Case volumes generally aligned with broader national epidemiological trends, such that locations assessed earlier during the outbreak were generally experiencing lower levels of incidence than those assessed later. For example, location A is a small, geographically dispersed yet interconnected community where case investigators and contact tracers were trusted members of the community, often going door-to-door to elicit contacts and provide care for patients with COVID-19. However, many locations, specifically those with large and more dense populations, relied on telephone calls to reach cases and elicit contacts. Another example of a programmatic difference was that location C adopted a test-out-of-quarantine policy before this assessment^30^; this policy likely contributed to the high proportion of contacts who underwent SARS-CoV-2 testing. Because of these differences, it is not appropriate to directly compare performance by locations.

Despite media reports of slow SARS-CoV-2 test turnaround times through the summer of 2020, HDs in this assessment received results quickly after specimen collection. To effectively reduce SARS-CoV-2 transmission, contact notification should occur within 6 days of exposure^31^; most of the HDs in this assessment met that threshold. To improve timeliness, locations C, H, and M adopted the practice of notifying household contacts during index case interviews of the need to quarantine.

Timely collection and communication of relevant information are essential for performing contact tracing. We observed that all 14 HDs in this assessment, regardless of the sophistication of their data collection and informatics systems, faced hurdles.^32,33^ These challenges included missing and incomplete data, paper-based records, disparate digital databases without common identifiers, and insufficient personnel to maintain, improve, or implement new systems. Most data systems were rapidly designed and were primarily intended to prioritize individual patient care and follow-up, rather than produce summary data describing the HD’s case investigation and contact tracing performance. A dedicated team of 3 persons, on average, worked 1 week to gather and summarize findings at each HD. Such an effort is not sustainable for HDs wishing to do ongoing self-assessment, particularly when local COVID-19 incidence is dynamic and increasing. However, for HDs seeking more systematic and ongoing evaluation, this report contributes standardized contact tracing performance metrics that could help HDs assess performance.

Contact tracing for other diseases, such as sexually transmitted infections and tuberculosis, typically rely on in-person interviews to develop rapport.^10,19,20^ However, most COVID-19 interviews occur over the telephone, are more time-sensitive, and do not have the benefit of multiple encounters with HD staff to establish trust. This trust is needed for COVID-19 contact tracing to overcome barriers such as concerns about subjecting contacts to quarantine and the perception that there is no tangible benefit to contacts. Educating communities about the importance of contact tracing, such as the Answer the Call campaign,^34^ is one strategy to help persons with COVID-19 feel more comfortable about having confidential conversations with health care practitioners and HD staff.

Complementary strategies include wider uptake of digital contact tracing tools. These include opt-in smartphone applications that can automate exposure notifications,^35^ often within hours of receiving the positive test result, to help increase the number of contacts who are confidentially notified about their exposure. In addition, automated symptom monitoring tools and case management software can help eliminate the need for HD staff to make daily telephone calls to asymptomatic contacts,^36^ perhaps allowing them to reallocate that time for more productive case interviews. Prospects for 7- to 10-day quarantine periods combined with symptom monitoring and testing as an exit strategy might make quarantine more palatable.^37^ Finally, it is also important to acknowledge that many persons might require additional social support services (eg, food, separate housing, and lost wage support) to complete isolation or quarantine.

### Limitations

Our assessment has several limitations. First, case investigation and contact tracing activities are designed to encourage case isolation and contact quarantine. We could not directly assess these actual interventions. Therefore, the assessment should be viewed as an indirect assessment of the effectiveness of COVID-19 case investigation and contact tracing. A second limitation is that these results might not generalize to other locations or time periods. However, we were able to include HDs from all regions of the US, representing different population characteristics and experiencing different epidemic trajectories. Additionally, because only 4 locations submitted data on reporting contacts by race and ethnicity, our observations about similarities across racial subgroups should not be generalized. Future assessments efforts should include data stratified by race and ethnicity. Third, there is no established standard for the expected number of contacts each person should have. Our methods assumed that those who did not participate would have, on average, provided the same number of contacts as those who did participate; therefore, our estimated number of missed contacts may be biased. However, we believe that our estimated expected number of contacts was conservative because the number of contacts reported per case was low. Contact tracing studies of respiratory infectious diseases with longer infectious periods have observed an average of 11 contacts per case,^38^ and COVID-19 modeling studies suggest upward of 30 contacts per case.^6^ However, perhaps many of the persons with COVID-19 who named no contacts had truly isolated themselves, had fewer contacts as they became aware of the magnitude of the pandemic, or might have notified their contacts on their own, and those contacts chose to self-quarantine without requiring any HD involvement in the process. Fourth, the higher SARS-CoV-2 test positivity rates among named contacts might have been biased because these data were not routinely captured for all contacts. Testing of contacts, particularly asymptomatic contacts,^39^ might not have been readily available and could have been further confounded by changing recommendations regarding the importance of testing all contacts. Fifth, effective contact tracing is dependent on transmission intensity, as well as resources available. Estimating the number of the staff needed to adequately perform contact tracing was important for program planning and implementation. We sought to measure this, but because each location chose to manage and divide the contact tracing workload differently, standardizing these measurements was challenging and, ultimately, beyond the scope of this study. Future evaluations should seek to measure the associations between staffing levels, training, job experience, and the performance of contact tracing.

## Conclusions

Scaling up COVID-19 contact tracing capacity during the pandemic has put enormous strain on every HD in the US. If a HD does not have the capacity to interview the majority of its new cases, then suspending or scaling down contact tracing activities could enable these public health resources to be reallocated for mass vaccination and other mitigation strategies.^9^ When COVID-19 vaccination coverage increases and disease incidence decreases to manageable numbers, HDs will be better positioned to successfully reach and interview every person in the community with a positive SARS-CoV-2 test result. To end the epidemic, multiple strategies, including contact tracing, universal masking, physical distancing, and COVID-19 vaccination, should be harmonized to reduce global incidence of this disease.

## Notes

Supplement 1. **eFigure.** Stages and Metrics in Case Investigation and Contact Tracing Cascade **eTable 1.** Key Metrics in Case Investigation and Contact Tracing Cascade, June-October 2020 **eTable 2.** Proportion of Persons With COVID-19 Who Named No Contacts During Case Interview, by Race and Ethnicity

Supplement 2. COVID-19 Contact Tracing Assessment Team
