# Supplementary material for: COVID-19 Case Investigation and Contact Tracing in the US, 2020
Source: JAMA Netw Open. 2021 Jun 3;4(6):e2115850. doi: 10.1001/jamanetworkopen.2021.15850 (PMC8176334; doi:10.1001/jamanetworkopen.2021.15850)
Supplement: Supplement 1. — eFigure. Stages and Metrics in Case Investigation and Contact Tracing Cascade eTable 1. Key Metrics in Case Investigation and Contact Tracing Cascade, June-October 2020 eTable 2. Proportion of Persons With COVID-19 Who Named No Contacts During Case Interview, by Race and Ethnicity [file jamanetwopen-e2115850-s001.pdf]

# Supplemental Online Content

Lash RR, Moonan PK, Byers BL, et al; COVID-19 Contact Tracing Assessment Team. COVID-19 case investigation and contact tracing in the US, 2020. *JAMA Netw Open*. 2021;4(6):e2115850. doi:10.1001/jamanetworkopen.2021.15850

**eFigure.** Stages and Metrics in Case Investigation and Contact Tracing Cascade

**eTable 1.** Key Metrics in Case Investigation and Contact Tracing Cascade, June-October 2020

**eTable 2.** Proportion of Persons With COVID-19 Who Named No Contacts During Case Interview, by Race and Ethnicity

This supplemental material has been provided by the authors to give readers additional information about their work.

© 2021 Lash RR et al. *JAMA Network Open.*

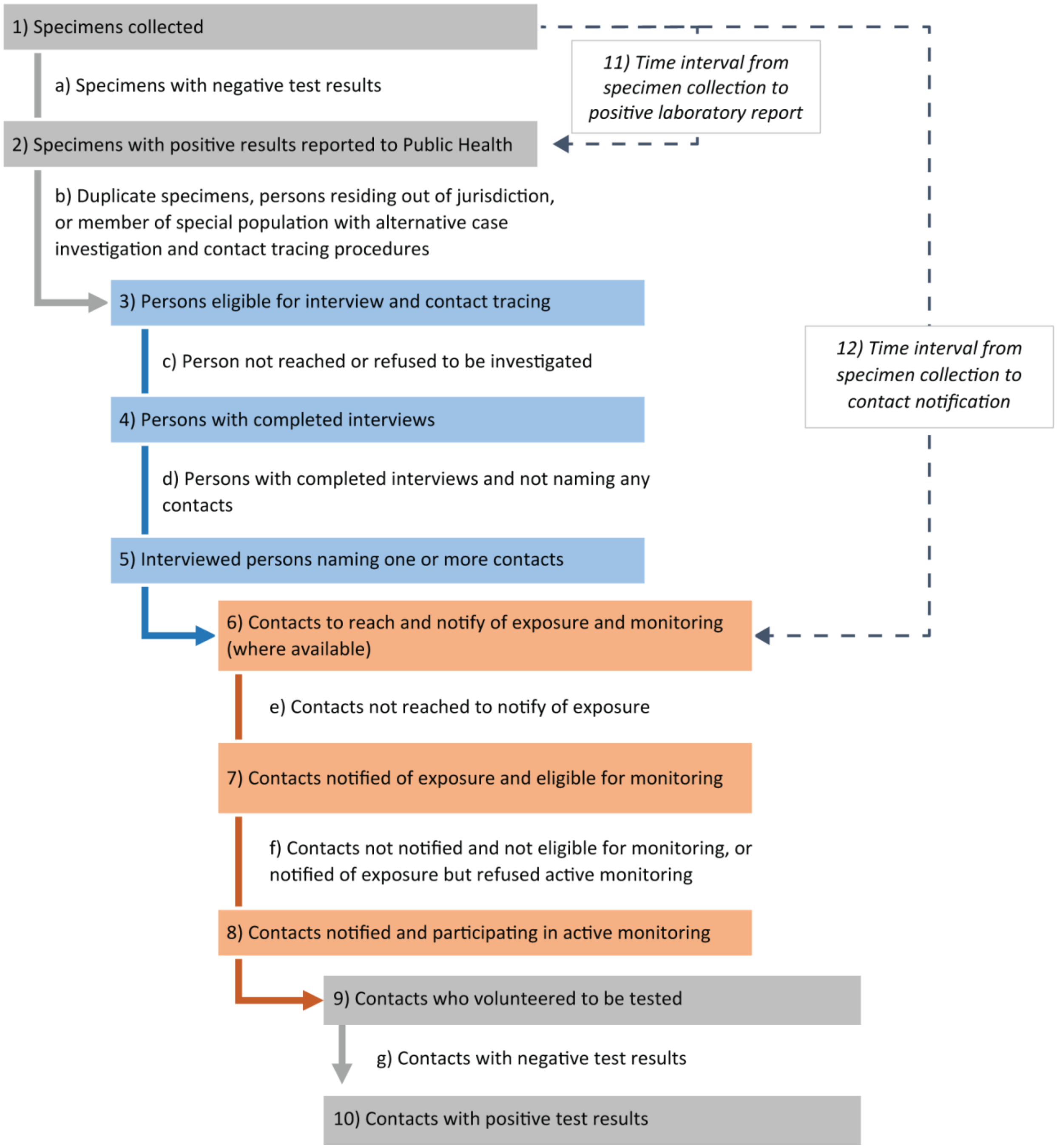
1) Specimens collected
a) Specimens with negative test results
2) Specimens with positive results reported to Public Health
11) Time interval from specimen collection to positive laboratory report
b) Duplicate specimens, persons residing out of jurisdiction, or member of special population with alternative case investigation and contact tracing procedures
3) Persons eligible for interview and contact tracing
12) Time interval from specimen collection to contact notification
c) Person not reached or refused to be investigated
4) Persons with completed interviews
d) Persons with completed interviews and not naming any contacts
5) Interviewed persons naming one or more contacts
6) Contacts to reach and notify of exposure and monitoring (where available)
e) Contacts not reached to notify of exposure
7) Contacts notified of exposure and eligible for monitoring
f) Contacts not notified and not eligible for monitoring, or notified of exposure but refused active monitoring
8) Contacts notified and participating in active monitoring
9) Contacts who volunteered to be tested
g) Contacts with negative test results
10) Contacts with positive test results

**eFigure.** Stages and Metrics in Case Investigation and Contact Tracing Cascade

The contact tracing cascade summarizes the stages required for successful case investigation and contact tracing. Stages have been grouped together according to the common denominator for the respective stage: diagnostic testing for SARS-CoV-2 among the general population (grey), investigation of persons diagnosed with SARS-CoV-2 infection (blue), notification and monitoring of contacts (orange), and diagnostic testing for SARS-CoV-2 among contacts (grey).

© 2021 Lash RR et al. *JAMA Network Open.*

**eTable 1. Key Metrics in Case Investigation and Contact Tracing Cascade, June-October 2020**

| Locations | Cases | | | Contacts | | |
|---|---|---|---|---|---|---|
| | **Total Reported** No. | **Interviewed** No. (%) | **Named any Contacts** No. (%) | **Total Identified** No. | **Notified of Exposure** No. (%) | **Agreed to Monitoring** No. (%) |
| A | 40 | 40 (100%) | 40 (100%) | 117 | 62 (53%) | 62 (100%) |
| B[23] | 589 | 584 (99%) | 382 (65%) | 1,146 | 602 (53%) | 552 (92%) |
| C | 146 | 144 (99%) | 125 (87%) | 404 | 382 (95%) | 75 (20%) |
| D | 137 | 136 (99%) | 86 (63%) | 359 | 341 (95%) | 336 (99%) |
| E | 718 | 655 (91%) | 289 (44%) | 712 | 604 (85%) | NA |
| F | 479 | 405 (85%) | 358 (88%) | 1,418 | 982 (69%) | 805 (82%) |
| G | 7,041 | 5,823 (83%) | 5,084 (87%) | 10,927 | 6,645 (61%) | 6,154 (93%) |
| H | 5,087 | 3,961 (78%) | 2,392 (60%) | 6,068 | 5,499 (91%) | NA |
| I[23] | 7,116 | 5,514 (77%) | 2,890 (52%) | 13,401 | 10,070 (75%) | 9,815 (97%) |
| J | 493 | 369 (75%) | 68 (14%) | 173 | 155 (90%) | NA |
| K | 10,563 | 7,820 (74%) | 3,651 (47%) | 11,569 | 6,773 (59%) | 5,177 (76%) |
| L | 10,757 | 5,228 (49%) | 1,425 (27%) | 2,848 | 2,474 (87%) | 2,472 (100%) |
| M | 22,032 | 10,290 (47%) | 7,284 (71%) | 24,190 | 17,909 (74%) | 8,177 (46%) |
| N | 8,987 | 2,962 (33%) | 631 (7%) | 1,507 | 816 (54%) | 720 (88%) |
| Total | 74,185 | 43,931 (59%) | 24,705 (33%) | 74,839 | 53,314 (71%) | 34,345 (46%) |

[23] Lash RR, Donovan CV, Fleischauer AT, et al. COVID-19 contact tracing in two counties—North Carolina, June-July 2020. MMWR Morb Mortal Wkly Rep. 2020;69:1360-1363.

© 2021 Lash RR et al. *JAMA Network Open.*

**eTable 2. Proportion of Persons With COVID-19 Who Named No Contacts During Case Interview, by Race and Ethnicity.**

| Characteristic | Location B, June 2020 | | | Location J, June 2020 | | | Location M, October 2020 | | | Location N, July–August 2020 | | |
|---|---|---|---|---|---|---|---|---|---|---|---|---|
| | **Interviewed** | **Named no contacts** | **PR** (95% CI[a]) | **Interviewed** | **Named no contacts** | **PR** (95% CI[a]) | **Interviewed** | **Named no contacts** | **PR** (95% CI[a]) | **Interviewed** | **Named no contacts** | **PR** (95% CI[a]) |
| Race — No. (%) | 589 | 202 (34) | | 364[b] | 258 (71) | | 1857[b] | 1419 (76) | | 2317[b] | 1803 (78) | |
| White | 531 | 180 (34) | Reference | 119 | 89 (75) | Reference | 758 | 588 (78) | Reference | 758 | 588 (78) | Reference |
| Black | 30 | 11 (37) | 1.1 (0.7–1.8) | 1 | 1 (100) | NA | 795 | 605 (76) | 1.0 (0.9–1.0) | 795 | 605 (76) | 0.9 (0.9–1.0) |
| Asian | 0 | 0 | NA | 0 | 0 | NA | 64 | 48 (75) | 1.0 (0.8–1.1) | 64 | 48 (75) | 1.0 (0.8–1.1) |
| Other and missing | 28 | 11 (39) | 1.2 (0.7–1.9) | 244 | 188 (77) | 1.0 (0.9– 1.2) | 240 | 178 (74) | 1.0 (0.9–1.0) | 700 | 562 (80) | 1.0 (1.0–1.1) |
| Ethnicity — No. (%) | 589 | 202 (34) | | 364[b] | 278 (76) | | 2962[b] | 2331 (79) | | 2624[b] | 2097 (80) | |
| Non-Hispanic | 314 | 114 (36) | Reference | 89 | 70 (79) | Reference | 1240 | 966 (78) | Reference | 1240 | 966 (78) | Reference |
| Hispanic | 273 | 87 (32) | 0.9 (0.7–1.1) | 272 | 206 (76) | 1.0 (0.8–1.1) | 555 | 425 (77) | 1.0 (0.9–1.0) | 555 | 425 (77) | 1.0 (0.9–1.0) |
| Missing | 2 | 1 (50) | NA | 3 | 2 (67) | NA | 1167 | 940 (81) | 1.0 (1.0–1.1) | 829 | 706 (85) | 1.1 (1.0–1.1) |

[a] CI denotes confidence interval. PR denotes prevalence ratio.

[b] This number is smaller than reported in Table S2, because not all individuals reached for case interview provided their race or ethnicity

© 2021 Lash RR et al. *JAMA Network Open.*
