# Supplementary material for: COVID-19 Case Investigation and Contact Tracing in the US, 2020
Source: JAMA Netw Open. 2021 Jun 3;4(6):e2115850. doi: 10.1001/jamanetworkopen.2021.15850 (PMC8176334; doi:10.1001/jamanetworkopen.2021.15850)
Supplement: Supplement 2. — COVID-19 Contact Tracing Assessment Team [file jamanetwopen-e2115850-s002.pdf]

Supplemental Online Content: Collaborators

*Indicates required information. Only first name, last name, and suffix will appear in PubMed.

***Group Name(s):** COVID-19 Contact Tracing Assessment Team							
***First Name and Middle Initial(s)**	***Last Name**	***Suffix (eg, Jr, III)**	Academic Degrees	Institution	Location (city, state/province, country)	Role or Contribution, eg, chair, principal investigator	Group (if more than 1 Group listed in the byline) and/or Subgroup (eg, Steering Committee)
Joshua L.	Clayton		PhD	South Dakota Department of Health	Pierre, South Dakota		
Heather Bailey	Forbes		MPH	Gwinnett, Newton, and Rockdale County Health Departments	Lawrenceville, Georgia		
Nick	DeLuca		PhD	US Centers for Disease Control and Prevention	Atlanta, Georgia		
Maryam	Haddad		PhD	US Centers for Disease Control and Prevention	Atlanta, Georgia		
Gibbie	Harris		MSPH	Mecklenburg County Public Health, Charlotte, North Carolina	Charlotte, North Carolina		
Susan	Hayes		MEd	Randolph County Public Health, Asheboro, North Carolina	Asheboro, North Carolina		
Blake	Hendrickson		MPH	Nebraska Department of Health and Human Services	Lincoln, Nebraska		
Margaret A.	Honein		PHD	US Centers for Disease Control and Prevention	Atlanta, Georgia		
Matthew	Hogben		PhD	US Centers for Disease Control and Prevention	Atlanta, Georgia		
Amanda	Jones		MPH	Vermont Department of Health	Burlington, Vermont		
Catherine J.	Knott		MPH	Vermont Department of Health	Burlington, Vermont		
Richard L.	Leman		MD	Oregon Health Authority	Portland, Oregon		

Supplemental Online Content: Collaborators

*Indicates required information. Only first name, last name, and suffix will appear in PubMed.

***First Name and Middle Initial(s)**	***Last Name**	***Suffix (eg, Jr, III)**	Academic Degrees	Institution	Location (city, state/province, country)	Role or Contribution, eg, chair, principal investigator	Group (if more than 1 Group listed in the byline) and/or Subgroup (eg, Steering Committee)
Stephen A.	McCurdy		MD, MPH	Marin County Division of Public Health	San Rafael, Califronia		
Alana	McGrath		MPH	Marin County Division of Public Health	San Rafael, Califronia		
Zack S.	Moore		MD	North Carolina Department of Health and Human Services	Raleigh, North Carolina		
Dustin	Ortbahn		MPH	South Dakota Department of Health	Pierre, South Dakota		
Sai	Paritala		PharmD, M	Nebraska Department of Health and Human Services	Lincoln, Nebraska		
Eric S.	Pevzner		PhD	US Centers for Disease Control and Prevention	Atlanta, Georgia		
Rebecca A.	Pierce		RN, PhD	Oregon Health Authority	Portland, Oregon		
Pratima L.	Raghunathan		PhD	US Centers for Disease Control and Prevention	Atlanta, Georgia		
Catherine	Rains		MPH	Springfield-Greene County Health Department, Springfield, MO	Springfield, Missouri		
Dale A.	Rose		PhD	US Centers for Disease Control and Prevention	Atlanta, Georgia		
Tom	Safranek		MD	Nebraska Department of Health and Human Services	Lincoln, Nebraska		
Bhavani	Sathya		MPH	New Jersey Department of Health	Trenton, New Jersey		
Christina G.	Tan		MD	New Jersey Department of Health	Trenton, New Jersey		

Supplemental Online Content: Collaborators

*Indicates required information. Only first name, last name, and suffix will appear in PubMed.

***First Name and Middle Initial(s)**	***Last Name**	***Suffix (eg, Jr, III)**	Academic Degrees	Institution	Location (city, state/province, country)	Role or Contribution, eg, chair, principal investigator	Group (if more than 1 Group listed in the byline) and/or Subgroup (eg, Steering Committee)
Melanie	Taylor		MD	US Centers for Disease Control and Prevention	Atlanta, Georgia		
Phoebe G.	Thorpe		MD, MPH	US Centers for Disease Control and Prevention	Atlanta, Georgia		
Henry	Walke		MD	US Centers for Disease Control and Prevention	Atlanta, Georgia		
Andee	Weisbeck		MPH	Gwinnett, Newton, Rockdale County Health Departments	Lawrenceville, Georgia		
